# The impact of the ‘open’ workspace on human collaboration

**DOI:** 10.1098/rstb.2017.0239

**Published:** 2018-07-02

**Authors:** Ethan S. Bernstein, Stephen Turban

**Affiliations:** 1 Harvard Business School, Boston, MA, USA; 2 Harvard University, Cambridge MA, USA

**Keywords:** interaction, transparency, collaboration, communication, spatial boundaries, collective intelligence

## Abstract

Organizations’ pursuit of increased workplace collaboration has led managers to transform traditional office spaces into ‘open’, transparency-enhancing architectures with fewer walls, doors and other spatial boundaries, yet there is scant direct empirical research on how human interaction patterns change as a result of these architectural changes. In two intervention-based field studies of corporate headquarters transitioning to more open office spaces, we empirically examined—using digital data from advanced wearable devices and from electronic communication servers—the effect of open office architectures on employees' face-to-face, email and instant messaging (IM) interaction patterns. Contrary to common belief, the volume of face-to-face interaction decreased significantly (approx. 70%) in both cases, with an associated increase in electronic interaction. In short, rather than prompting increasingly vibrant face-to-face collaboration, open architecture appeared to trigger a natural human response to socially withdraw from officemates and interact instead over email and IM. This is the first study to empirically measure both face-to-face and electronic interaction before and after the adoption of open office architecture. The results inform our understanding of the impact on human behaviour of workspaces that trend towards fewer spatial boundaries.

This article is part of the theme issue ‘Interdisciplinary approaches for uncovering the impacts of architecture on collective behaviour’.

## 1. Introduction

Boundaries between ‘us’ and ‘them’ have long captured human interest. Yet even as social scientists continue to study the value of a vast array of boundaries [1], in an era in which the nature of work is changing [2–4], managers and organizational scholars have increasingly framed boundaries as barriers to interaction that ought to be spanned [5–8], permeated [9] or blurred [10] to increase collaboration. In the most physically salient and concrete example, ‘spatial boundaries’ [11] at work—such as office or cubicle walls—are being removed to create open ‘unbounded’ offices in order to stimulate greater collaboration and collective intelligence. Does it work?

Prior theory is divided—and empirical evidence mixed—on the effect that removing spatial boundaries has on human behaviour in the space previously within those boundaries (e.g. [12,13]). On the one hand, sociological theory presents a strong argument that removing spatial boundaries to bring more people into contact should *increase* collaboration and collective intelligence. The notion that propinquity, or proximity, predicts social interaction [14]—driving the formation of social ties and therefore information exchange and collaboration—is one of the most robust findings in sociology [15,16]. It has been observed in contexts as diverse as the US Congress [17,18], nineteenth-century boarding houses [19], college dormitories [14], laboratories [20], co-working spaces [21] and corporate buildings [22]. When spatial boundaries—such as walls—are removed, individuals feel more physically proximate, which, such theory suggests, should lead to more interaction. Such interaction is a necessary foundation for collective intelligence—a form of distributed intelligence that arises from the social interaction of individuals [23] and that predicts, more so than the intelligence of individual members, a group's general ability to perform a wide variety of tasks [24–26]. Much like the swarm intelligence observed among cognitively simple agents such as social insects and other animals [27–29], collective intelligence for groups of humans requires interaction [30]. If greater propinquity drives greater interaction, it should generate greater collaboration and collective intelligence.

On the other hand, some organizational scholars, especially social psychologists and environmental psychologists, have shown that removing spatial boundaries can *decrease* collaboration and collective intelligence. Spatial boundaries have long served a functional role at multiple levels of analysis, helping people make sense of their environment by modularizing it [31], clarifying who is watching and who is not, who has information and who does not, who belongs and who does not, who controls what and who does not, to whom one answers and to whom one does not [32]. This school of thought, like theories of organizational design and architecture [29], assumes that spatial boundaries built into workspace architecture support collaboration and collective intelligence by mitigating the effects of the cognitive constraints of the human beings working within them. Like social insects which swarm within functionally-determined zones ‘partitioned’ by spatial boundaries (e.g. hives, nests or schools) [29], human beings—despite their greater cognitive abilities—may also require boundaries to constrain their interactions, thereby reducing the potential for overload, distraction, bias, myopia and other symptoms of bounded rationality. Research as far back as the foundational Hawthorne Studies [33,34] shows that being walled off can therefore increase interaction within the separated group [33]. Similarly, subsequent workplace design research (for reviews, see [35–38])—though mixed in its findings—suggests that open offices can reduce certain conditions conducive to collaboration and collective intelligence, including employee satisfaction [39,40], focus [41–44], psychological privacy [45,46] and other affective and behavioural responses [40,41,43,47,48]. Such negative psychological effects of open offices conceivably may lead to less, not more, interaction between those within them [49], reducing collaboration and collective intelligence.

To our knowledge, no prior study has directly measured the effect on *actual interaction* that results from removing spatial boundaries to create an open office environment. Past workplace design research, rather than directly and objectively measuring behaviours, has relied heavily on survey-based or activity-log methodologies, which provided self-reported measures, or on social observation studies, which provided an observer's subjective interpretation of human interactions. Several decades ago, when much of the workplace design research was conducted, measuring actual interaction patterns of individuals at work in both traditional and open office environments would have been prohibitively difficult, but new ‘people analytics’ technology has made it quite feasible.

Using two field studies of organizations transforming their office architecture by removing spatial boundaries to become more open, we empirically measure the effect on interaction, carefully tracking face-to-face (F2F) interaction before and after the transition with wearable sociometric devices [50,51] that avoid the imprecise and subjective survey-based self-reported measures typical of previous office collaboration studies [52,53]. We also measure two digital channels of interaction—email and instant messaging (IM) [54–56]—using information from the organizations' own servers.

In the first study, we focus on the most basic set of empirical questions: what is the effect of transitioning from cubicles to open workspaces on the overall *volume* and *type* of interaction, with what implications for organizational performance based on the company's own performance management system? In the second study, we replicate the first study's results and then consider two more-targeted empirical questions: how does spatial *distance* between workstations moderate the effect of transitioning from cubicles to open workspaces and how do individual employee interaction *networks*, both F2F and electronic, change differentially? While the first study considers interactions involving *individuals*, the second considers interactions for *dyads* (both sides of the interaction), allowing a more precise but limited investigation of the effects.

## 2. Study 1

The first empirical study, a quasi-field experiment [57,58], was conducted at the global headquarters of OpenCo1,^1^ a Fortune 500 multinational. In a so-called war on walls, OpenCo1 decided to use the latest open office workstation products to completely transform the wall-bounded workspaces in its headquarters so that one entire floor was open, transparent and boundaryless.

The redesign—which required people to move from assigned seats on their original floor to similarly assigned seats on a redesigned floor of the same size—affected employees in functions including technology, sales and pricing, human resources (HR), finance, and product development, as well as the top leadership. Of those people, a cluster of 52 (roughly 40%) agreed to participate in the experiment. A comparison of HR data for participants and nonparticipants provided no evidence of nonresponse bias. Because of the nature of office space, all employees moved from the old space to the redesigned space at the same time, so the experiment was structured with an interrupted time-series design [58].

**Figure 1.**
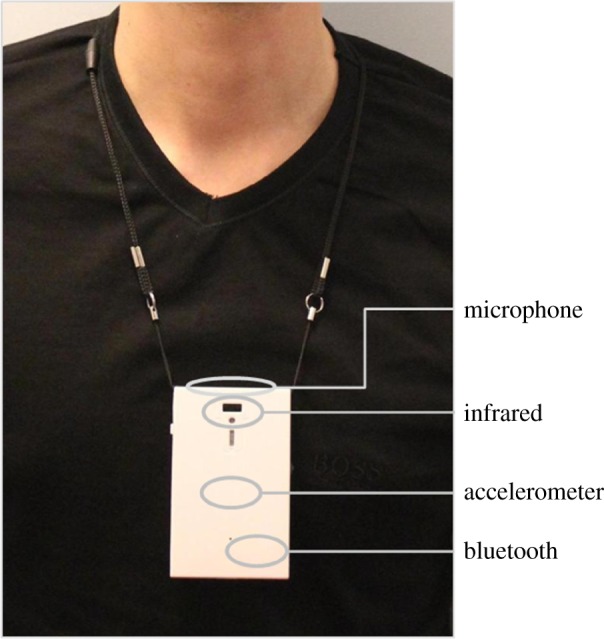
Sociometric badge. (Online version in colour.)

To capture a full, data-rich picture of interaction patterns both before and after the boundaries were removed, participants were asked to wear a sensor, known as a sociometric badge [59], that recorded, in great detail, their F2F interactions: an infrared (IR) sensor captured whom they were facing (by making contact with the other person's IR sensor), microphones captured whether they were talking or listening (but not what was said), an accelerometer captured body movement and posture, and a Bluetooth sensor captured spatial location (figure 1). All sensors recorded time-stamped data in 10 ms intervals. Based on prior research using these sociometric badges [50], an F2F interaction was recorded when three conditions were met: two or more badges (i) were facing each other (with uninterrupted infrared line-of-sight), (ii) detected alternating speaking, and (iii) were within 10 m of each other. The interaction ended when any of the three criteria ceased to be true for more than 5 s. While these criteria were based on precedent from significant prior use of sociometric badges, sensitivity analysis showed the results to be robust to reasonable alternative assumptions (including shorter distances in 1 m increments, different lag times before concluding an interaction, and different speaking patterns). This F2F data was combined with email and IM data for the same time periods, collected from the company's servers, to create a full picture of these professionals' interactions before and after the redesign.

Data were collected in two phases: for 15 workdays (three weeks) before the redesign and, roughly three months later, for 15 workdays after the redesign. Three-week data collection windows were chosen as a balance between the organization's desire to minimize the burden of the research study on its employees and our need to control for the possibility of idiosyncratic daily and weekly variations in employee schedules. The three-month gap between phases was chosen for two reasons. First, work at OpenCo1's global headquarters followed quarterly cycles, so a three-month gap allowed us to conduct the two data-collection phases at the same point in the quarter. Second, it allowed just over two months of adjustment after the move, enough for people to have settled into their new environment but not so much that the work they did could have changed much.

The dataset included 96 778 F2F interactions, 84 026 emails (18 748 sent, 55 012 received, 9755 received by cc and 511 received by bcc) and 25 691 IMs (consisting of 221 426 words). The most straightforward and conservative empirical strategy for analysing the intervention was to simply aggregate and then compare pre-intervention and post-intervention volumes:2.1
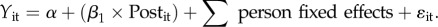


*Y*_it_, the dependent variable, is the amount of interaction—F2F or electronic—where ‘i’ is the individual in question and ‘t’ is the phase (pre- or post-redesign). Post_it_ is an indicator variable that equals 1 if the interaction occurred after the redesign. The main estimation used ordinary least-squares (OLS) regressions with person fixed effects, although all results were robust to the exclusion of person fixed effects. Standard errors were corrected for autocorrelation and clustered by individual [60]. If the redesign increased F2F interaction, we should see a positive and significant *β*_1_— the coefficient reported in the ‘Post’ column of table 1—when *Y*_it_ is F2F interaction (the first row of table 1). More generally, in table 1, the effect on a particular kind of interaction due to the transition to more open architecture is reported in the ‘post’ column, where a negative number indicates reduced interaction and a positive number indicates increased interaction.

**Table 1.** Impact of open offices on interaction at OpenCo1. Models are OLS with person fixed effects and with standard errors clustered by individual in parentheses. Coefficients represent minutes of face-to-face (F2F) interaction, number of email messages or IM messages, or number of words in IM between a member of the study and all others at work during the period of the study. *p<0.05; **p<0.01; ***p<0.001.

type of interaction	post	constant	obs.
***volume:***			
*F2F interaction* minutes of F2F interaction time (indicated by proximity of individuals combined with spoken words by at least one party)	−3774* (1607)	5266*** (1136)	104
*email interaction (sent)* total number of emails sent by participants to other participants	66*** (19)	118*** (13)	104
*email interaction (received: To)* total number of emails received by participants from other participants, where the recipient appeared in the ‘To:’ field	78*** (21)	394*** (15)	104
*email interaction (received: cc)* total number of emails received by participants from other participants, where the recipient appeared in the ‘Cc:’ field	27*** (8)	66*** (6)	104
*email interaction (received: bcc)* total number of emails received by participants from other participants, where the recipient appeared in the ‘Bcc:’ field	−1 (1)	6*** (1)	104
*IM interaction (number of messages)* total number of instant messages sent by participants to other participants	99** (30)	147*** (21)	104
*IM interaction (cumulative word count of messages)* total number of words sent in instant messages by participants to other participants	850*** (218)	1140*** (154)	104

### (a) Study 1 results

#### (i) Volume of interaction

Although OpenCo1's primary purpose in opening up the space had been to increase F2F interactions, the 52 participants now spent 72% *less* time interacting F2F. Prior to the redesign, they accumulated 5266 min of interaction over 15 days, or roughly 5.8 h of F2F interaction per person per day. After the redesign, those same people accumulated only 1492 min of interaction over 15 days, or roughly 1.7 h per person per day.

Even though everyone on the floor could see everyone else all the time (or perhaps *because* they could), virtual interaction replaced F2F interaction in the newly boundaryless space. After the redesign, participants collectively sent 56% (66) more emails to other participants over 15 days, received 20% (78) more emails from other participants, and were cc'd on 41% (27) more emails from other participants. (For the received and cc'd volumes, emails sent are counted once for each recipient.) Bcc: activity, which was low in volume and limited to a small subset of individuals, did not significantly change. IM message activity increased by 67% (99 more messages) and words sent by IM increased by 75% (850 more words). Thus—to restate more precisely—in boundaryless space, electronic interaction replaced F2F interaction.

#### (ii) Performance outcome

Should we be concerned about these effects? One indication of the meaningfulness of this shift in behaviour was its effect on performance. In an internal and confidential management review, OpenCo1 executives reported to us qualitatively that productivity, as defined by the metrics used by their internal performance management system, had declined after the redesign to eliminate spatial boundaries. Consistent with research on the impact of a decline in media richness on productivity [54,55] and on the particular challenges of email [61], it is not necessarily surprising that productivity declined due to a substitution of email for F2F interaction. What is surprising is that more open, transparent architecture prompted such a substitution.

## 3. Study 2

Given the findings from Study 1, another organization was recruited to further this research. Our goal was to conduct a conceptual replication of the first study with a longer time window. This second empirical study was also a quasi-field experiment at a Fortune 500 multinational and was conducted at the global headquarters of OpenCo2.^2^ At the time of the study, OpenCo2 was in the process of a multi-year headquarters redesign, which—as in Study 1—involved a transformation from assigned seats in cubicles to similarly assigned seats in an open office design, with large rooms of desks and monitors and no dividers between people's desks.

We again collected F2F data using sociometric badges and email data from company servers, this time for 100 employees from a single floor, which was roughly 45% of the employees on that floor. As in Study 1, data were collected in two phases: for eight weeks starting three months prior to the redesign of this particular floor and for eight weeks starting two months after the redesign. But for this study, we also collected detailed data on the participants; namely, three employee attributes—gender, team assignment and role—and one architectural attribute—desk location. In the first phase, desks were in cubicles, so seats were roughly 2 m apart and directly adjacent to one another. In the second phase, seats still lay roughly 2 m apart and directly adjacent to one another, but were grouped at undivided and unwalled tables of six to eight. Seat location allowed us to calculate the physical distance between dyads of employee workstations before and after the redesign, such that we could include physical distance, as well as the other employee attributes, as control variables. The OpenCo2 dataset included 63 363 min of F2F interaction and 25 553 emails, all generated by 1830 dyads—those with interaction—of the 100 employees involved. Mindful of Study 1's consistent results across multiple forms of electronic communication, Study 2 only collected email data to measure electronic interaction. The empirical strategy was similar:3.1
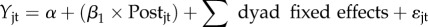
and3.2
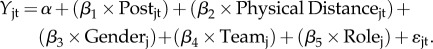


In equation (3.1), as in equation (2.1), *Y*_jt_, the dependent variable, is the amount of interaction, F2F or electronic. However, because the physical-distance control variable was dyadic, *Y*_jt_ must also be specific to a particular dyad ‘j’ (rather than to an individual ‘i’, as in Study 1). As in Study 1, ‘t’ refers to the phase (pre- or post-redesign). Post_jt_ is an indicator variable that equals 1 if the dyadic interaction occurred after the redesign. In equation (3.2), we investigate specific control variables—characteristics of each dyad—rather than just dyad fixed effects. Physical Distance_jt_ is the distance between the dyad's workstations, measured as the shortest walking path (in metres). Gender_j_, Team_j_ and Role_j_ are indicator variables that equal 1 if the two individuals in the dyad were of the same gender, on the same team, or in the same role, and equal 0 otherwise. The main estimation used OLS regressions with either dyad fixed effects (2) or distance, gender, team and role controls (3). Standard errors of the coefficients were corrected for autocorrelation and clustered by dyad [60]. If the redesign increased F2F interaction, we should see a positive and significant *β*_1_—the coefficient reported in the ‘post’ row of table 2—when *Y*_it_ is F2F interaction. More generally, in table 2, we report the effect of the transition to open architecture on particular types of interaction in the ‘post’ row, where a negative number indicates reduced interaction and a positive number indicates increased interaction. For the control variables, we report the coefficient for the entire sample without regard to whether the office architecture involved cubicles or open spaces, as our purpose in including those variables is to remove gender, team and role effects from the variable of interest, Post. For example, the significant and positive coefficient for Team means that those on the same team communicated more than those on different teams (for both cubicles and open spaces), and the significant and positive coefficient for Role means that those in the same role communicated more than those in different roles (for both cubicles and open spaces).

**Table 2.** Impact of open offices on interaction at OpenCo2. Models are OLS with standard errors clustered by dyad in parentheses. Models 1 and 3 include dyad fixed effects. In Models 1 and 2, coefficients represent minutes of F2F interaction between a particular dyad during the period of the study. In Models 3 and 4, coefficients represent number of emails between a particular dyad during the period of the study. *p<0.05, **p<0.01, ***p<0.001.

type of interaction	1	2	3	4
	F2F with fixed effects	F2F with controls	email with fixed effects	email with controls
***change in volume:***				
*post* 0 if before redesign, 1 if after	−12.79*** (1.39)	−9.81*** (1.27)	1.24*** (0.31)	1.54*** (0.32)
*physical distance* walking distance (in metres) between desks	−0.01 (0.02)	−0.07*** (0.02)	−0.00 (0.01)	−0.01 (0.01)
*gender* 0 if different genders, 1 if same		2.08 (1.37)		0.08 (1.02)
*team* 0 if different teams, 1 if same		41.02*** (2.53)		33.86*** (1.80)
*role* 0 if different roles, 1 if same		9.59*** (1.91)		3.12* (1.42)
*constant*	17.99*** (1.27)	14.63*** (1.47)	5.75*** (0.28)	3.07*** (0.85)
observations	3660	3660	3660	3660

### (a) Study 2 results

#### (i) Volume of interactions

As a result of the redesign, 643 dyads decreased their F2F interaction and 141 dyads increased it. At the same time, 222 dyads decreased their email interaction and 374 dyads increased it. Like OpenCo1, OpenCo2 had hoped, by opening up the space, to increase F2F interactions, but the results did not bear this out. The 100 employees—or 1830 dyads—we tracked spent between 67% (Model 1, 12.79/17.99) and 71% (Model 2, 9.81/14.63) *less* time interacting F2F. Instead, they emailed each other between 22% (Model 3, 1.24/5.75) and 50% (Model 4, 1.54/3.07) more.

As one might suspect, dyads on the same team or with the same role communicated more, both F2F and by email, relative to dyads on different teams or in different roles. Gender, in contrast, had no significant effect on the volume of either form of interaction. Physical distance did show a small inverse effect on F2F interaction (Model 2): the nearer the two workstations, the more F2F interaction. This effect was notable both for its small size relative to the size of the effect of the open office and for the fact that it was limited to F2F interaction (not email). We investigate this in further detail next.

#### (ii) The effect of physical distance on F2F versus email

Model 2 of table 2 shows that the effect of physical distance on F2F interaction is small—and the effect on email insignificant—relative to that of openness. The relatively small effect of distance on F2F interaction was surprising given that repeated studies have shown that people talk more to those who are physically closer to them [62,63]. When others are physically proximate, it is easier to be aware of them [64], start conversations with them [64,65], unexpectedly encounter or overhear them [66], and manage their impressions of our collaborative work behaviour [67]. Nonetheless, our review of these prior studies found none that directly measured interaction volumes, and thus perhaps—while present—the effect of distance on F2F interaction may be far more minimal than previously thought.

Table 2, however, does not allow us to compare the relative effects of physical distance on F2F interaction and on email interaction. To do so, we used a latent space model called the Latent Position Clustering Model [68] to take into account clustering and to control for other covariates. We find that physical distance affected F2F interaction twice as much as it did email interaction. As a robustness check, we used several machine learning algorithms, such as a Random Forest, to see if changes in F2F networks prompted by changes in physical distance predicted changes in email networks. Across all models, we find that F2F networks and email networks respond very differently to changes in the built environment, with changes in one type of network failing to predict changes in the other.

This variance between the adaptation of F2F and electronic networks in response to a change in physical space is an important finding for future research on collaboration and collective intelligence. In several notable cases, past research has relied on email alone [69,70] to study topics ranging from the Enron debacle to the relationship between office layout and interaction, basing claims about F2F interaction on findings from electronic interaction data. Our finding that changes in workplace design affect electronic and F2F interaction networks differently (and, on some measures, in opposite directions) should make future researchers wary of using one network as a proxy for the other.

## 4. Discussion

We began with a specific research question: does removing spatial boundaries at work to create open, unbounded offices increase interaction? Our two empirical field studies were consistent in their answer: open, unbounded offices reduce F2F interaction with a magnitude, in these contexts, of about 70%. Electronic interaction takes up at least some of the slack, increasing by roughly 20% to 50% (as measured by ‘To:’ received email).

Many organizations, like our two field sites, transform their office architectures into open spaces with the intention of creating more F2F interaction and thus a more vibrant work environment. What they often get—as captured by a steady stream of news articles professing the death of the open office [71–73]—is an open expanse of proximal employees choosing to isolate themselves as best they can (e.g. by wearing large headphones [74]) while appearing to be as busy as possible (since everyone can see them). Recent studies [75] and earlier research [40,41,43,47,48] have investigated the self-reported dissatisfaction of employees in open offices, but to our knowledge, we are the first to empirically study the direct behavioural impact of open office space on the volume of F2F and electronic interaction. Our results support three cautionary tales.

First, transitions to open office architecture do not necessarily promote open interaction. Consistent with the fundamental human desire for privacy [76] and prior evidence that privacy may increase productivity [32,45], when office architecture makes everyone more observable or ‘transparent’, it can dampen F2F interaction, as employees find other strategies to preserve their privacy; for example, by choosing a different channel through which to communicate [39]. Rather than have an F2F interaction in front of a large audience of peers, an employee might look around, see that a particular person is at his or her desk, and send an email.

The second caution relates to the impact of a transition to open office architecture on collective intelligence. We still have much to learn about how collective intelligence works [77], as we borrow from and distinguish parallel work on swarm intelligence among social insects and some other animals. While the earliest work assumed open spaces would promote collective intelligence among humans, our findings support more recent work that has begun to suggest otherwise. Kao & Couzin, in modelling the presence of multiple cues and the possibility of observing them, find that intermediate (rather than maximal) levels of cues produce higher levels of collective intelligence [78]. We see a close relationship between our finding that open, ‘transparent’ offices may be overstimulating and thus decrease organizational productivity and Kao & Couzin's demonstration that finitely bounded, and often small, group size maximizes decision accuracy in complex, realistic environments. Similarly, recent collective intelligence work suggests that, like our open offices, too much information from social data can be problematic, partly because of challenges focusing attention [74,79], but also for reasons that extend to more general functions of human cognition. For example, by connecting human cognition and collective intelligence with the behaviour of eusocial insects, Toyokawa *et al*. found that richness in social information was detrimental to collective intelligence outcomes, with performance being best when social learning opportunities were constrained [80]. Similarly, in a study involving human subjects, Bernstein *et al*. found that intermittent rather than constant social influence produced the best performance among humans collectively engaged in complex problem solving [81]. As we are reminded in Hight & Perry's article on collective intelligence and architectural design, ‘collective intelligence is not simply technical, but also explicitly social, political, and by extension, professional’ [2, p. 6]. Our findings empirically reinforce their caution that the relationship between architectural design and collective intelligence extends beyond technical considerations.

The third caution is that transitions to open office architecture can have different effects on different channels of interaction. In our studies, openness decreased F2F interaction with an associated increase in email interaction. In the digital age, employees can choose from multiple channels of interaction [54] and a change in office architecture may affect that choice.

Complementing prior research on media richness suggesting that substituting email for F2F interaction can lower productivity [53], our studies highlight two other consequences. First, because fundamentally different mechanisms drive F2F and email interaction, the physical propinquity that redesigned offices seek to achieve has a direct effect only on F2F interaction, not on email, yet drives interaction from F2F to email. Adopting open offices, therefore, appears to have the perverse outcome of reducing rather than increasing productive interaction. Second, F2F and email networks differ. Although prior studies have investigated one or the other [56,82], none has empirically linked F2F and email network interaction to discern how good a proxy one is for the other. We find that they are poor proxies for each other. Therefore, an intervention that redirects interaction from one network to another, like the open office redesigns studied here, not only changes the channel of interaction, but also skews *whom* a person interacts with. That can have profound consequences for how—and how productively—work gets done.

In summary, because the antecedents of human interaction at work go beyond proximity and visibility, the effects of open office architecture on collaboration are not as simple as previously thought. While it is possible to bring chemical substances together under specific conditions of temperature and pressure to form the desired compound, more factors seem to be at work in achieving a similar effect with humans. Until we understand those factors, we may be surprised to find a reduction in F2F collaboration at work even as we architect transparent, open spaces intended to increase it.

## Endnotes

1 OpenCo1 is a pseudonym for the corporation's real name, which has been anonymized.

2 OpenCo2 is a pseudonym for the corporation's real name, which has been anonymized.

## Data accessibility

We are unable to provide open access to our data owing to their sensitive nature and the nondisclosure and confidentiality agreements that surround them. Please contact the corresponding author for more information.
